# The complete mitochondrial genome and phylogenetic analysis of *Amusium pleuronectes*

**DOI:** 10.1080/23802359.2020.1772691

**Published:** 2020-06-05

**Authors:** Lijie Yao, Hongwei Yu, Yaran Liu, Yuehuan Zhang, Yuli Li

**Affiliations:** a MOE Key Laboratory of Marine Genetics and Breeding, College of Marine Life Sciences, Ocean University of China, Qingdao, China; b Laboratory for Marine Biology and Biotechnology, Pilot National Laboratory for Marine Science and Technology, Qingdao, China; c Key Laboratory of Tropical Marine Bio-resources and Ecology, South China Sea Institute of Oceanology, Chinese Academy of Sciences, Guangzhou, China

**Keywords:** *Amusium pleuronectes*, mitochondrial genome, phylogenetic analysis

## Abstract

*Amusium pleuronectes*, commonly known as the Asian moon scallop, is widely distributed in Indo-Pacific coasts. In this study, the complete mitogenome sequence of *A. pleuronectes* (18,044 bp) is reported, which represents the first mitogenome from the *Amusium* genus. This mitogenome contains 13 protein-coding genes, 2 rRNAs, and 22 tRNA genes, showing similar mitogenome features for most marine bivalves. Phylogenetic analysis reveals that within the family Pectinidae, the genus *Amusium* is closely related to the genus *Argopecten*. The mitogenome of *A. pleuronectes* provides a valuable resource for further advancing the understanding of bivalve phylogeny and evolution.

*Amusium pleuronectes*, commonly known as the Asian moon scallop, is widely distributed in Indo-Pacific coastal areas (Minchin 2003) and characterized by beautiful two-colored shells. Except for high economic value, the Asian moon scallop is often used as a biological monitor to evaluate the impact of human activities on the marine environment (Siriprom and Limsuwan 2009). However, genetic information for this species remains scarce. Recently, complete mitogenome has been considered as an effective tool for phylogeny and phylogeography studies (Curole and Kocher 1999; Saccone et al. 1999). In this study, we report the sequencing and assembly of the complete mitogenome of *A. pleuronectes*, which is the first mitogenome of the genus *Amusium*.

The example of *A. pleuronectes* was derived from Tung Ping Chau Bay near Tung Chung village (Guangdong province, China, 22.4907°N, 114.5801°E) at a depth of 7–8 m. All soft tissues were preserved in liquid nitrogen after sampling. Total DNA was extracted using the phenol/chloroform/isoamyl alcohol method (Sambrook et al. 1989). The DNA sample was deposited at the Key Laboratory of Marine Genetics and Breeding (Ministry of Education), Ocean University of China (Specimen code: OUC-MGB-2018-AP-08). Whole genomic sequencing of the individual sample was performed using the Illumina NovaSeq 6000 sequencing platform. The mitogenome of *A. pleuronectes* was *de novo* assembled using NOVOPlasty (Dierckxsens et al. 2017), gene information of which was retrieved by the MITOS software (Bernt et al. 2013).

The complete circular mitogenome of *A. pleuronectes* was 18,044 bp in length. Thirteen protein-coding genes (PCGs), 2 ribosomal RNA genes, and 21 transfer RNA genes were annotated. The 13 conserved PCGs were cytochrome oxidase subunit (I, II, and III), NADH dehydrogenase subunit (1, 2, 3, 4, 5, 6, and 4 L), and ATP synthase subunit (6, and 8), which was similar to the reported mitochondrial genomes of most marine bivalve mollusks (Liu et al. 2019; Ma et al. 2019; Liu et al. 2020). The base composition of *A. pleuronectes* was A = 22.22%, T = 38.60%, G = 25.55%, and C = 13.63%. The overall AT content (60.82%) was higher than the GC content (39.18%). The small subunit ribosomal RNA (12S rRNA) and large subunit ribosomal RNA (16S rRNA) were annotated with sizes of 968 bp and 1,420 bp, respectively. The length of 22 tRNAs ranged from 63 bp to 72 bp. The whole mitogenome sequence has been deposited in GenBank under the accession number of MT419374.

Previous studies of the phylogeny of the family Pectinidae were mostly based on the sequence information of few genes (Serb 2016; Smedley et al. 2019). In this study, a mitogenome-level phylogenetic tree was constructed based on all the available scallop mitogenomes, including *A. pleuronectes* and 11 other scallop species (from six genera of Pectinidae), as well as two outgroup species (Figure 1). The phylogenetic tree was constructed using the neighbor-joining (NJ) algorithm with 1000 bootstrap trials. The phylogeny tree indicated that the genus *Amusium* represented by *A. pleuronectes* was closely related to the genus *Argopecten* as reported by previous studies (Serb 2016; Smedley et al. 2019). The *A. pleuronectes* mitogenome provides a valuable resource for further advancing our understanding of bivalve phylogeny and evolution.

**Figure 1.**
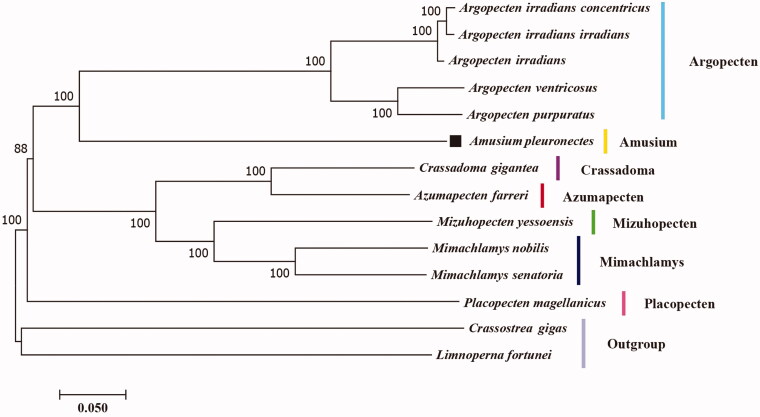
The phylogenetic tree of *A. pleuronectes* and 13 other bivalves based on complete mitochondrial genomes. The accession numbers of downloaded sequences are as follows: *Argopecten irradians concentricus* (KT161259.1), *Argopecten irradians irradians* (NC_012977.1), *Argopecten irradians* (NC_009687.1), *Argopecten venteicosus* (KT161261.1), *Argopecten purpuratus* (NC_027943.1), *Crassadoma gigantea* (MH016739.1), *Azumapecten farreri* (NC_012138.1), *Mizuhopecten yessoensis* (NC_009081.1), *Mimachlamys nobilis* (NC_011608.1), *Mimachlamys senatoria* (NC_022416.1), *Placopecten magellanicus* (NC_007234.1), *Crassostrea gigas* (NC_001276.1), and *Limnoperna fortunei* (NC_028706.1).

## Data Availability

The data that support the findings of this study are openly available in GeneBank at [https://www.ncbi.nlm.nih.gov/nuccore/MT419374.1/], reference number [Accession number: *MT419374*].
